# Changes in strength performance of highly trained athletes after COVID-19

**DOI:** 10.1371/journal.pone.0308955

**Published:** 2024-09-19

**Authors:** Jie Cao, Shengtao Yang, Jinhao Wang, Peng Zhang

**Affiliations:** Professional Sports Research Center, Shanghai Research Institute of Sports Science (Shanghai Anti-Doping Agency), Shanghai, China; La Trobe University, AUSTRALIA

## Abstract

**Introduction:**

This study aimed to explore the impact of COVID-19 on strength performance in highly trained athletes.

**Method:**

A force plate was employed to measure squat jump height (SJH), counter-movement jump height (CMJH), and drop jump reactive strength index (DJRSI) in 27 highly trained athletes before infection, and at one week, two weeks, and four weeks post-recovery. Additionally, an Isometric Mid-thigh Pull (IMTP) test was conducted to record maximum isometric strength (MIS) and the rate of force development of the initial phase (RFD 0–50; RFD 0–100). Repeated measures analysis of variance was utilized to compare variations in these indicators across different time points.

**Results:**

One week post-recovery, SJH (-7.71%, P = 0.005), CMJH (-9.08%, P < 0.001), DJRSI (-28.88%, P < 0.001), MIS (-18.95%, P < 0.001), RFD 0–50 (-64.98%, P < 0.001), and RFD 0–100 (-53.65%, P < 0.001) were significantly lower than pre-infection levels. Four weeks post-recovery, SJH (-2.08%, P = 0.236), CMJH (-3.28%, P = 0.277), and MIS (-3.32%, P = 0.174) did not differ significantly from pre-infection levels. However, DJRSI (-11.24%, P = 0.013), RFD 0–50 (-31.37%, P = 0.002), and RFD 0–100 (-18.99%, P = 0.001) remained significantly lower than pre-infection levels.

**Conclusion:**

After COVID-19, highly trained athletes exhibited a significant reduction in maximum strength, explosive strength, reactive strength, and initial phase force generation capability. By four weeks post-recovery, their maximum and explosive strength had returned to near pre-infection levels, yet their reactive strength and initial phase force generation capability remained significantly impaired.

## Introduction

Coronavirus disease (COVID-19), caused by the severe acute respiratory syndrome coronavirus 2 (SARS-CoV-2), is an acute infectious disease characterized by high transmissibility, affecting a broad segment of the global population [1, 2]. The Omicron variant of SARS-CoV-2 became the predominant strain worldwide at the beginning of 2022 [3], and it has been the main cause of coronavirus infections in China from 2022 to 2023 [4]. Research has shown that SARS-CoV-2 can cause considerable harm to both the nervous system and skeletal muscles [1, 5–8]. Common post-infection symptoms include fatigue, headaches, dizziness, and muscle and joint pain [5–10], with some patients exhibiting pronounced sarcopenia [11]. These neurological and muscular impairments have the potential to influence strength performance in humans. Current research has reported that uninfected athletes have experienced a degree of negative impact on their physiological traits and strength performance after a period of training cessation due to isolation [12, 13, 56]. However, research on the impact of COVID-19 on strength performance is still very limited, particularly regarding its effects on various different aspects of athletes’ strength performance.

Strength performance (such as maximum strength, explosive strength, and reactive strength) is one of the most important factors influencing an athlete’s sports performance [12, 16, 23, 28, 58, 62]. Given the scarcity of research on the effects of COVID-19 on the strength performance of athletes, definitive conclusions regarding the decline in athletes’ strength post-infection and its subsequent impact on competitive performance have yet to be established. The issue of how to train and prepare for competitions following recovery from the virus remains a challenge for both coaches and athletes. With SARS-CoV-2 transitioning into a phase of coexistence with humanity, the risk of reinfection and the potential for new mutations triggering another pandemic persist. Considering the scarcity of research on the impact of COVID-19 on athletes’ strength, in 2023, we conducted this study on strength performance among athletes from Shanghai who were infected with the SARS-CoV-2 (Omicron variant) both before and after their infection. The purpose of this research was to assess the impact of COVID-19 and the subsequent cessation of training on various aspects of the athletes’ strength(including maximum strength, explosive strength, reactive strength, the rate of strength development), and the characteristics of strength recovery within the month following their recovery. The findings of this study may offer valuable insights that can inform the training and competitive strategies for athletes who have recovered from COVID-19 in the current context. Furthermore, the study provides a reference for potential future resurgences of SARS-CoV-2 and other similar viral infections, ensuring that athletes are better prepared to manage the impact on their performance and health.

## Methods

### 1. Study design

This study was conducted in compliance with the Declaration of Helsinki, and the protocol was approved by the Ethics Committee of the Shanghai Research Institute of Sports Science (Shanghai Anti-Doping Agency) (approval number LLSC20230001). The minimum sample size is calculated using GPower software, and the minimum sample size required for this study is 15. The athletes in this study come from Shanghai Elite Sports Training Administrative Center. On average, they participated in systematic special training 8 years ago (From athletes in the local amateur sports school to participate in special training to the present time), and currently they are engaged in special training for about 40 hours per week (6.5 to 7 hours per day, 6 days per week). All the athletes participated in national competitions in China. According to McKay et al’s classification criteria for athlete levels, the athletes in this study were highly trained [14].

Before the study, Participants were informed about the procedures and the associated potential risks. All Participants or parents of Participants signed written informed consent. In this study, the squat jump (SJ) was utilized to measure the concentric explosive strength of the participants’ lower limbs [15]. The counter-movement jump (CMJ) was employed to assess the explosive strength during a slow Stretch Shortening Cycle (SSC) [16, 17]. The drop jump (DJ) test served as a measure for reactive strength [18]. Additionally, the isometric mid-thigh pull (IMTP) test was administered to determine the maximum strength of the lower limbs. The rate of force development (RFD) during the IMTP served as an evaluation of the Participants’ ability to quickly generate force at the start of the force production [19, 20]. Before the COVID-19 (Omicron variant) outbreak in Shanghai, 108 athletes from the Shanghai Elite Sports Training Administrative Center participated in a battery of strength tests, which included SJ, CMJ, DJ, and IMTP. After the outbreak, three additional testing sessions were organized for athletes who met the following criteria: 1) Their first-time infection with the novel coronavirus, 2) Moderate disease severity as per the World Health Organization’s classification system for COVID-19 [2], and 3) Availability of pretest data for the SJ, CMJ, DJ, and IMTP within two months before infection. From these, a group of twenty-seven athletes (14 males and 13 females) from various sports such as modern pentathlon, fencing, boxing, and judo were selected for the study between January 5 and February 25, 2023. The basic characteristics of the Participants are detailed in Table 1.

**Table 1:** Basic characteristics of participants.

	Age (y)	Height (cm)	Body mass (kg)	BMI/(kg·m^-2^)	Training years (y)
Male(n = 14)	20.2±2.3	183.1±4.0	73.8±9.4	21.9±2.1	8.3±1.3
Female(n = 13)	21.0±2.4	172.3±5.5	61.4±7.8	20.6±1.8	8.8±1.9
Overall(n = 27)	20.6±2.4	177.9±7.2	67.8±10.6	21.3±2.1	8.5±1.6

Note:Training years = the number of years since an athlete has been training specifically at a local amateur sports school.

Before the onset of infection, participants were tested for SARS-CoV-2 nucleic acid three times per week, on Mondays, Wednesdays, and Fridays. After contracting the virus, they were tested daily. The date of the first positive SARS-CoV-2 nucleic acid test was marked as the start of the infection, with the length of time positive results were defined as the infection stage. On average, the infection duration for participants was about one week (6.93±1.98 days). The day a SARS-CoV-2 nucleic acid test turned negative was considered the first day of recovery. After infection, The first test was scheduled approximately one week post-recovery (7.84±1.21 days), the second test was two weeks post-recovery (15.51±2.26 days), and the third test occurred four weeks post-recovery (29.81±2.32 days). In this study, the recovery training for athletes after infection follows the same guideline, which was formulated by the Shanghai Elite Sports Training Administrative Center and spans a total of 4 weeks. The intensity division in the guideline adheres to the American College of Sports Medicine (ACSM) classification for cardiorespiratory and resistance training intensities, categorizing training intensity into low, moderate, and high levels based on indicators such as heart rate, rating of perceived exertion (RPE), and percentage of One Repetition Maximum (1RM) [21]. According to the guideline,during the period from the athlete’s infection to one week after recovery, no endurance, strength, or specific training is conducted. In the second week of recovery, athletes were only allowed to begin low-intensity endurance training. In the third week of recovery, athletes start with low-intensity strength and specific training. In the fourth week of recovery, athletes engage in moderate-intensity endurance, strength, and specific training. The timing of the testing and the training content and intensity are shown in Table 2.

**Table 2:** Test time and training regimens.

Stages of Infection	Schedule for Testing	Training Content and Intensity
**Pre-infection**	**Pre-test**:Within two months before contracting COVID-19	**Normal training**
**Infection stage** (6.93±1.98days)	**No testing**	**No training**
**1st week post-recovery**	**1st test**:About 1 week post-recovery (7.84±1.21 days)	**No training**
**2nd week post-recovery**	**2nd test**:About 2 weeks post-recovery (15.51±2.26 days)	**Low-intensity endurance training:**4–6 sessions of 30 minutes of jogging at a speed of 6–8 km/h, with a heart rate below 60% of HRmax and an RPE score below 10.
**3rd week post-recovery**	**No testing**	**Low-intensity endurance, strength and specific training:**Three 45-minute jogging sessions are conducted at a speed of 7-9km/h, with a heart rate below 65% HRmax, and an RPE score below 12. Strength training intensity is below 50% of 1RM, with a total of four sessions, each lasting 60–90 minutes, including bodyweight exercises such as squats, bench presses, pull-ups and push-ups, without plyometric exercises. Specific training mainly includes isolated upper and lower limb technical movement training and slow field movement training, without competition-style specific training.
**4th week post-recovery**	**3rd test**:About 4 weeks post-recovery (29.81±2.32 days)	**Moderate-intensity endurance, strength and specific training:**Three endurance training sessions lasting 45–60 minutes, with a running speed of 9-12km/h, heart rate between 65%-75% HRmax. Four strength training sessions with an intensity of 50%-70% of 1RM, each lasting about 90 minutes, including the previous week’s exercises plus jumping and agility training. Specific training mainly includes technical movement training, field-specific movement training, and continuous technical training at moderate intensity, such as continuous striking technique training in boxing.

### 2. Test methods

#### 2.1 SJ, CMJ and DJ

A three-dimensional force plate (Kistler, model 9287CA) was used to measure the SJ, CMJ, and DJ. The data acquisition rate was set to 1000Hz, and the collected data was then imported into the MARS analysis software for calculating force, height, and other relevant metrics. Before each test, participants completed a 25-minute warm-up and had their weight measured. The test procedures were clearly explained to the Participants, after which they performed a trial jump. A minimum of one-minute interval was maintained between each test to prevent fatigue. To minimize the potential placebo effects that could result from athletes’ infections, we consistently stress and encourage athletes to give their full effort across all tests in order to maximize their strength performance. Participants were required to perform at least three jumps. If a participant’s third jump resulted in a higher height than their second jump, additional jumps were conducted until there was a decrease in height compared to the previous jump. Jump height (H) was determined using the takeoff time method, where the vertical jump take off time (T) was identified as the moment when the vertical ground reaction force dropped below 10 N. Using the acceleration due to gravity, which is g = 9.8 m/s², the jump height was calculated using the formula H = 0.5g(T/2)² [22]. The specific criteria and metrics for these three tests are described below:

(1) SJ

Participants placed their hands on their hips, just above the iliac crests, and then squatted down, bending their knees to a 90° angle, which they held for a count of three seconds. They then performed a maximal effort vertical jump without any preparatory reverse motion. During the jump, participants kept their hands by their sides, maintained an upright posture in the air, and landed with their feet naturally positioned on the force plate. The test metrics was the squat jump height (SJH).

(2) CMJ

Participants placed their hands on their hips, above the iliac crest, and stood still for a count of three seconds. They then initiated a counter-movement jump, which involved a downward squat to an angle between 90° and 135° at the knee joints before jumping upwards with maximal effort. During the execution of the jump, participants were instructed not to move their arms and to keep their body in an upright position while in the air, landing with their feet naturally touching down on the platform. The test metrics was the counter-movement jump height (CMJH).

(3) DJ

Participants stood on a 30 cm jump box with one leg while extending the other leg out in front of their body. They then dropped onto the force platform, ensuring both feet made contact simultaneously, and jumped with maximal effort as quickly as possible. A touchdown time of 250 milliseconds or less was considered a valid trial [23]. Throughout the test, participants kept their hands on their hips, fully extended their bodies during the flight phase, and stepped down steadily from the force-measuring platform after landing. Two practice jumps were allowed before the actual testing began. A total of three tests were performed. The drop jump height (DJH) and drop jump contact time (DJCT) data were recorded. The drop jump reactive strength index (DJRSI) was obtained by dividing DJH by DJCT [23]. The DJRSI test data of the highest of the three tests was used for this study.

**Fig 1:**
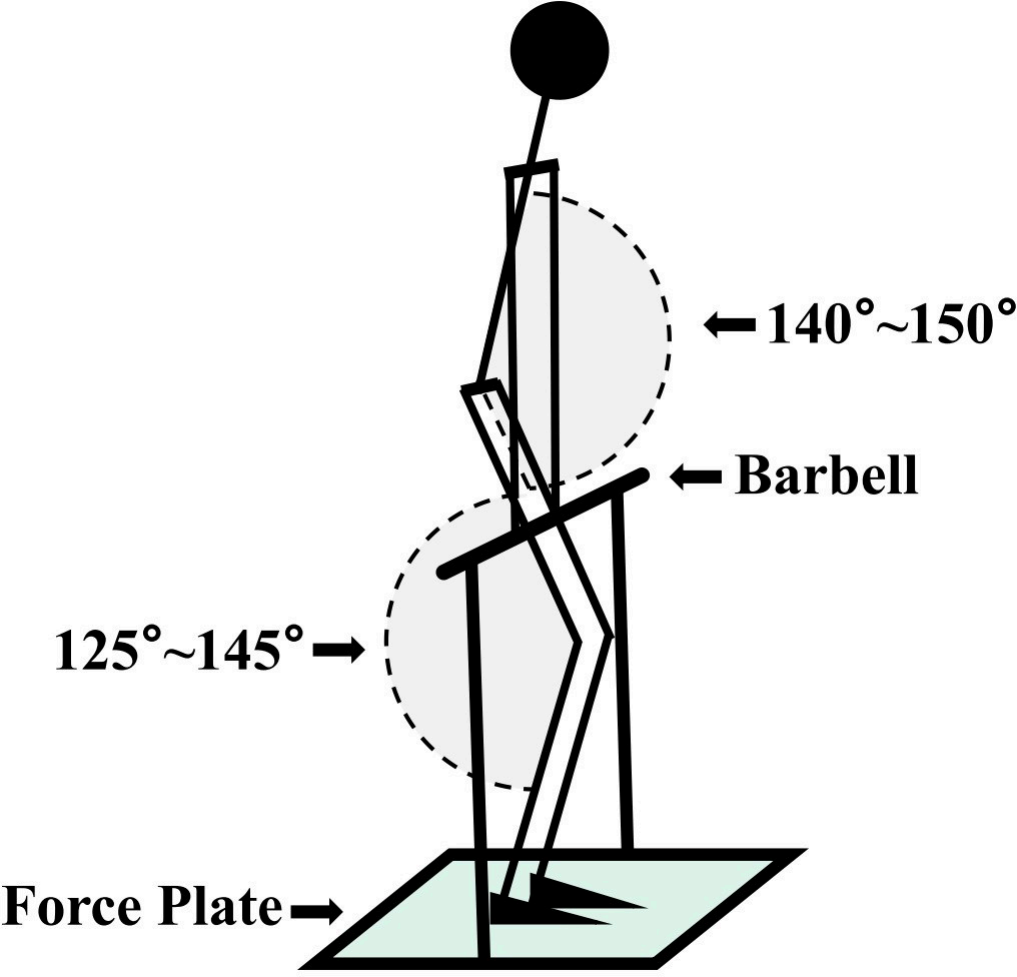
IMTP test.

**2.2 IMTP.** Data for the IMTP test were collected using a force plate set at a frequency of 1000 Hz. Participants began with a 25-minute warm-up before the test. Participants stand on the force plate with their feet positioned shoulder-width apart, with the barbell positioned at the midpoint of the thigh (the midpoint of the line connecting the patella and the anterior superior iliac spine), while maintaining the hip joint angle between 140°and 150°and the knee joint angle between 125°and 145°[24]. Participants keep their thighs close to the barbell, use lifting straps with both hands, and adopt a standard deadlift stance (Fig 1). During the first test, the grip width, stance width, and the angles of the hip and knee joints of the participants, as well as the height at which the barbell is fixed on the IMTP testing rig, are recorded. For all subsequent tests, the joint angles and body posture of the participants are kept consistent with those of the first test. To prepare, Participants performed pulls for 3 seconds at 50% and 70% of their perceived maximum force, with more than a minute of rest in between, before proceeding to the actual test after a 5-minute interval. The formal test commenced with a countdown from 3, 2, 1. Under the "start" command, the subject exerted maximum force for the shortest period of time, without any reverse movement, and pulled continuously for 5 seconds. This was repeated for three trials, with a 5-minute break between each. The test was considered valid if the difference in peak force between the three trials was less than 250 N [24–26]. The onset of muscle contraction was identified by a force increase exceeding five times the standard deviation of body weight fluctuation on the force plate [24, 25]. The isometric peak force was recorded as the highest average force within a 1-second window [19, 20]. The test with the highest peak force among the three tests was used as the data source. Maximum isometric strength (MIS) was calculated without body weight. the rate of force development during the initial 0–50 ms (RFD 0–50) and 0–100 ms (RFD 0–100) were calculated [26].

### 3. Data statistics and analysis

The data collected in this study were analyzed using SPSS 25.0 software. Outliers were identified using a Box Plot, and any data points that deviated from the mean by more than three times the standard deviation were excluded. 10 RFD data points from five athletes were excluded as they exceeded three times the standard deviation. The intraclass correlation coefficient (ICC) for indicators was calculated using the Two-way random model with the absolute agreement type. The ranges of ICC and coefficient of variation (CV) for the four tests of all indicators are as follows: SJ: ICC = 0.983–0.993, CV = 1.90%-2.35%; CMJ: ICC = 0.969–0.988, CV = 1.97%-2.51%; DJ: ICC = 0.962–0.990, CV = 2.05%-2.76%; MIS: ICC = 0.969–0.988, CV = 2.03%-2.49%; RFD 0–50: ICC = 0.802–0.883, CV = 5.56%-7.80%; RFD 0–100: ICC = 0.823–0.890, CV = 5.48%-7.12%. The ICC and CV results for the aforementioned indicators are all above the minimum acceptable standards for reliability in research (ICC >0.75 and CV <15%) [27–29]. A one-way repeated measures analysis of variance (ANOVA) was utilized to compare the variances of the test indicators across different time points, with a significance level set at P < 0.05. The Shapiro-Wilk method was employed to verify the normal distribution of the data. The data from this study was found to adhere to a normal distribution, and each variable was represented as the mean ± standard deviation (M±SD). Mauchly’s Test of Sphericity was used to determine whether the variables conformed to a spherical distribution, and the Greenhouse-Geisser method was applied to adjust for variables that did not meet the sphericity distribution. The Bonferroni method was used to adjust the test level, followed by pairwise comparisons. The effect size (η^2^) was calculated using SPSS, and according to Cohen’s classification of effect sizes [30], small effects were defined as 0.01 ≤ η^2^ < 0.06, medium effects as 0.06 ≤ η^2^ < 0.14, and large effects as η^2^≥ 0.14.

## Results

### 1. Changes of SJ and CMJ after COVID-19

As illustrated in Table 3, the main effect of time on SJH was significant, with a large effect size (η^2^ = 0.232). Further simple effect analysis indicated that SJH one week post-recovery was significantly lower than pre-infection (-7.71%, P = 0.005), and the SJH in the second week post-recovery was still significantly lower than that pre-infection (-4.23%, P = 0.007). By the fourth week post-recovery, SJH had significantly increased compared to the first week (+6.10%, P = 0.001), and there was no significant difference compared to pre-infection (-2.08%, P = 0.236).

**Table 3:** Changes of SJ and CMJ after COVID-19 (n = 27).

	Pre-infection	1 week post-recovery	2 weeks post-recovery	4 weeks post-recovery	*F*	*P*	η^2^
SJH(cm)	35.32 ± 6.28^bc^	32.60 ± 7.61^ad^	33.83 ± 6.82^a^	34.59 ± 7.18^b^	7.87	<0.001	0.232
Δ		-7.71% ± 6.22%	-4.23% ± 5.58%	-2.08% ± 4.78%			
CMJH(cm)	40.49 ± 7.00^bc^	36.81 ± 7.21^ad^	37.63 ± 8.26^ad^	39.16 ± 8.38^bc^	14.67	<0.001	0.361
Δ		-9.08% ± 5.63%	-7.08% ±7.14%	-3.28% ± 5.89%			

Values are mean ±SD,a = compared with Pre-infection, *P* < 0.05. b = compared with 1 week post-recovery, *P* < 0.05. c = compared with 2 weeks post-recovery, *P* < 0.05. d = compared with 4 weeks post-recovery, *P* < 0.05. Δ = 100% x (mean post-recovery ‐ mean pre-infection) / mean pre-infection. The η^2^ was calculated from SPSS. The following tables are the same.

The main effect of time on CMJH was also significant, with a large effect size (η^2^ = 0.361). Simple effect analysis revealed that CMJH one week post-recovery was significantly lower than pre-infection (-9.08%, P < 0.001), and similarly, CMJH two weeks post-recovery was significantly lower than pre-infection (-7.08%, P < 0.001). By the fourth week post-recovery, CMJH had significantly increased compared to the first and second weeks (+6.38%, P = 0.01; +4.06%, P = 0.001), with no significant difference from pre-infection (-3.28%, P = 0.277).

### 2. Changes of DJ after COVID-19

As shown in Table 4, the main effect of time on DJRSI was significant, with a large effect size (η^2^ = 0.694). Further analysis showed that DJRSI one and two weeks post-recovery were significantly lower than pre-infection (-28.88%, P < 0.001; -24.43%, P < 0.001). By the fourth week post-recovery, DJRSI had significantly increased compared to the first and second weeks (+24.8%, P < 0.001; +17.4%, P < 0.001), but it remained significantly lower than pre-infection (-11.24%, P = 0.013).

**Table 4:** Changes of DJ after COVID-19 (n = 27).

	Pre-infection	1 week post-recovery	2 weeks post-recovery	4 weeks post-recovery	*F*	*P*	η^2^
DJRSI (m**·**s^-1^)	1.644±0.358^bcd^	1.169±0.288^ad^	1.242±0.281^ad^	1.460±0.288^abc^	59.08	<0.001	0.694
Δ		-28.88% ± 11.27%	-24.43% ± 9.93%	-11.24% ± 10.57%			
DJH(cm)	29.35±5.67^bc^	23.82±4.75^ad^	24.97±4.85^ad^	27.99±4.88^bc^	32.98	<0.001	0.559
Δ		-18.84% ± 9.81%	-14.93% ± 8.61%	-4.64% ± 9.74%			
DJCT(s)	0.180±0.017^bcd^	0.209±0.035^a^	0.204±0.024^a^	0.195±0.027^a^	12.74	<0.001	0.329
Δ		+15.78% ±17.12%	+12.96% ± 11.74%	+7.93% ±10.25%			

The main effect of time on DJH was found to be significant, with a large effect size (η^2^ = 0.559). Simple effect analysis indicated that DJH during the first and second weeks post-recovery were both significantly lower compared to pre-infection (-18.84%, P < 0.001; -14.93%, P < 0.001). However, by the fourth week post-recovery, DJH had significantly improved compared to the first and second weeks post-recovery (+17.50%, P < 0.001; +12.09%, P < 0.001), and there were no significant differences compared with pre-infection (- 4.64%, P = 0.693).

The main effect of time on DJCT was statistically significant, with a large effect size (η^2^ = 0.329). Simple effect analysis revealed that DJCT during the first, second, and fourth weeks post-recovery were all significantly higher than the pre-infection (+15.78%, P = 0.001; +12.96%, P < 0.001; +7.93%, P = 0.008). Additionally, there were no significant differences in DJCT between the three post-recovery time points.

### 3. Changes of MIS after COVID-19

As shown in Table 5, the main effect of time on MIS was significant, with a large effect size (η^2^ = 0.493). Simple effect analysis indicated that MIS one and two weeks post-recovery were significantly lower than pre-infection (-18.95%, P < 0.001; -11.78%, P = 0.014). MIS in the second week post-recovery was significantly higher than in the first week (+8.84%, P = 0.002). MIS in the fourth week after recovery was significantly higher than that in the first week (+19.27%, P < 0.001) and the second week (+9.59%, P = 0.033), and there was no significant difference in MIS at week 4 post-recovery compared with pre-infection (-3.32%, P = 0.174).

**Table 5:** Changes of MIS after COVID-19 (n = 22).

	Pre-infection	1 week post-recovery	2 weeks post-recovery	4 weeks post-recovery	*F*	*P*	η^2^
MIS(N)	2041.50±207.53^bc^	1654.64±190.92^acd^	1801.09±178.86^abd^	1973.82±193.80^bc^	14.83	<0.001	0.497
Δ		-18.95% ± 5.32%	-11.78% ± 7.52%	-3.32% ± 3.93%			

### 4. Changes of RFD after COVID-19

As illustrated in Table 6, the main effect of time on RFD 0–50 was significant, with a large effect size (η^2^ = 0.661). Simple effect analysis showed that RFD 0–50 one and two weeks post-recovery were significantly lower than pre-infection (-64.98%, P < 0.001; -58.66%, P < 0.001). RFD 0–50 at four weeks post-recovery had significantly increased compared to weeks one and two post-recovery (+95.99%, P < 0.001; +66.00%, P = 0.001), but remained significantly lower than pre-infection (-31.37%, P = 0.002).

**Table 6:** Changes of RFD after COVID-19 (n = 22).

	Pre-infection	1 week post-recovery	2 weeks post-recovery	4 weeks post-recovery	*F*	*P*	η^2^
RFD 0–50 (N**·**s^-1^)	3225.05±955.54^bcd^	1129.32±411.21^ad^	1333.23±529.08^ad^	2213.32±705.47^abc^	29.09	<0.001	0.661
Δ		-64.98% ± 18.47%	-58.66% ± 19.64%	-31.37% ± 17.44%			
RFD 0–100 (N**·**s^-1^)	4427.05±994.85^bcd^	2051.95±838.37^acd^	2569.32±981.25^abd^	3586.55±1150.32^abc^	31.95	<0.001	0.680
Δ		-53.65% ± 17.92%	-41.96% ± 16.57%	-18.99% ± 16.08%			

The main effect of time on RFD 0–100 was also significant, with a large effect size (η^2^ = 0.680). Simple effect analysis indicated that RFD 0–100 one and two weeks post-recovery were significantly lower than pre-infection (-53.65%, P < 0.001; -41.96%, P < 0.001). RFD 0–100 at two weeks post-recovery had significantly increased compared to one week (+25.21%, P = 0.039), and by four weeks post-recovery, RFD 0–100 had significantly increased compared to weeks one and two (+74.79%, P < 0.001; +39.59%, P = 0.001), but still remained lower than pre-infection (-18.99%, P = 0.001).

## Discussion

The study found that athletes experienced a significant decrease in various aspects of strength performance following infection to COVID-19. Specifically, there was a noticeable reduction in concentric explosive strength (SJH: -7.71%), explosive strength under SSC (CMJH: -9.08%), maximum strength (MIS: -18.95%), reactive strength (DJRSI: -28.86%), and the ability to generate force in the initial phase (RFD 0–50:-64.98% & RFD 0–100:- 53.65%). The most significant reduction was observed in the initial phase force generation ability, followed by reactive strength and maximum strength, with explosive strength under SSC and concentric explosive strength showing a minor decrease.

After four weeks of recovery, the study found that the extent of restoration in various strength performances varied. Concentric explosive strength (97.92% of pre-infection levels), explosive strength within the SSC (96.72% of pre-infection levels), and maximum strength (96.68% of pre-infection levels) had almost returned to their pre-infection levels. In contrast, reactive strength (88.80% of pre-infection levels) and the capacity to generate force in the initial phase (68.63% and 81.01% of pre-infection levels for the first 50 ms and first 100 ms, respectively) remained significantly below their initial levels after four weeks of recovery. The recovery in these areas was notably slower than the other strength performances.

The structure and function of the nervous system and skeletal muscle are critical determinants of strength performance [31, 32]. The observed decline in strength performance among the athletes post-infection may be attributed to the damage inflicted by COVID-19 on the human nervous system and skeletal muscles and the interruption of training due to the illness. Research indicates that SARS-CoV-2 infection can cause significant damage to the nervous system. The virus can cross the blood-brain barrier and the trigeminal nerve pathway, infecting the central and peripheral nervous systems [33–35], leading to inflammation and demyelination of nerve cells [36, 37]. Post-infection, individuals often experience general fatigue, anosmia, ageusia, headaches, dizziness, and muscle and joint pain [8, 33, 37]. Reports suggest that 27% of athletes continue to experience these symptoms 28 days after infection, and a significant number, 38%, and 22%, suffer from long-term symptoms like fatigue and myalgia, respectively [7, 8, 38–40], highlighting the persistent impact of SARS-CoV-2 on the nervous system. Furthermore, SARS-CoV-2 infection is associated with autonomic nerve dysfunction, manifesting as neuropathic pain, palpitations, loss of Achilles tendon reflex, and distal muscle weakness and atrophy, among other symptoms [41, 42]. These symptoms could further impair an individual’s strength performance. The collective findings indicate that SARS-CoV-2 infection can significantly impair nervous system function, potentially contributing to the reduced strength performance observed in athletes following infection [43, 44].

Reports suggest that SARS-CoV-2 can inflict substantial damage on skeletal muscle. Upon infection, cytokines induced by SARS-CoV-2 and proinflammatory signaling molecules trigger pathological alterations in the skeletal muscle tissue [43, 44]. Specifically, interleukin-1β (IL-1β) and tumor necrosis factor (TNF-α) impede muscle cell growth by inhibiting the proliferation and differentiation of satellite cells, which are essential for muscle fiber growth [43, 44]. Additionally, interferon-γ (IFN-γ) and interleukin-6 (IL-6) can directly cause muscle fibrin hydrolysis and diminish protein synthesis, leading to a decrease in skeletal muscle mass [45–49]. IL1β and IL-6 may also provoke fibrosis within muscle cells, compromising the muscle’s force-generating capacity [50, 51]. Post-infection, a prevalence of symptoms such as muscle weakness (60%-72%), myalgia (38%-71%), arthralgia (31%-61%), and sarcopenia (28%-37%) is commonly observed [7, 36, 52–57]. Further, studies document that a considerable proportion of patients continue to experience muscle weakness (25%), myalgia (22%), and sarcopenia (16%) over extended periods, ranging from 30 days to 1 year after recovery [8, 54, 55]. These findings underscore that SARS-CoV-2 can significantly impair the structure and function of skeletal muscle, potentially constituting another crucial factor contributing to the substantial decrement in strength performance observed among athletes in this study following infection.

Short-term detraining (up to 4 weeks) can negatively affect strength performance [58]. The cessation of training can significantly influence the voluntary force production of human skeletal muscles, resulting from the interaction between neural and morphological factors. These factors include muscle cross-sectional area, architecture, muscle fiber type, tendon properties, and the neural drive to the spinal-motor pool [59]. It is reported that the cross-sectional area of muscle fibers in athletes can be notably reduced within 2 to 3 weeks of detraining [60, 61]. Following a 14-day halt in training, there is a significant decrease in both the maximum isometric strength and maximum eccentric strength of the athletes’ knee joint (-7% and -12%, respectively), with a concomitant reduction of approximately 10% in surface electromyography activity [61]. A marked decrease in power output during swimming (-13.6%) was observed after four weeks of detraining [62]. Moreover, detraining can detrimentally affect tendon performance, leading to reduced stiffness in the lower limbs [63–65], which can influence an athlete’s strength performance to a certain degree. In this study, the athletes did not perform any training from the time of infection to the first week of recovery (15.77±2.21 days after infection). They only undertook low-intensity aerobic training during the second week of recovery (22.44±2.26 days after infection). The duration of strength cessation reached 3–4 weeks, similar to that in the above study. Therefore, we speculate that detraining due to COVID-19 may also negatively impact athletes’ strength performance to a certain extent.

In this study, the capacity to rapidly generate force in the initial phase is highly dependent on the nerve’s ability to quickly recruit muscle fibers, which is closely linked to neuromuscular function [66, 67] and is also associated with the stiffness of the lower limb tendons [18, 67]. The findings revealed that the RFD within the first 50 ms post-infection experienced the most significant decrease (-64.98%), followed by RFD within the first 100 ms (-53.65%), DJ (-28.86%, with an average push-off time of 193 ms), CMJ (-9.08%, with an average push-off time of 316 ms), and SJ (-7.71%, with an average push-off time of 403 ms). The overall trend indicated that the shorter the duration of force generation, the more pronounced the decline in strength performance. This substantial decrease in strength performance over a short period is likely related to the novel coronavirus’s impairing effect on the nerve’s rapid muscle recruitment capability. As per the research reports cited, the novel coronavirus can cause damage to the central nervous system, peripheral nerves, and autonomic nerves, leading to inflammation of nerve cells, demyelination, headaches, dizziness, general fatigue, and various autonomic nervous system disorders and symptoms [8, 33, 36–38]. This damage may notably reduce the frequency of action potential release from motor neurons, slow the conduction velocity of action potentials in nerves, and impair the function of the neuromuscular junction. Consequently, this could slow the nerve’s recruitment rate to muscles, leading to a decreased RFD in the initial period and, ultimately, a more significant reduction in strength performance over a brief period. Furthermore, the athletes in this study experienced two weeks of detraining after infection (with an average of 15.77 days of complete detraining), significantly increasing their bed rest time and drastically reducing the time spent on fast-moving and jumping activities. This reduction in activity may have decreased the stiffness of the lower limbs and slowed the force transmission velocity in the muscles and tendons, contributing to the more substantial decrease in strength performance observed over a short time frame.

The findings from this study reveal that after a four-week recovery period, both reactive strength and the rate of force development (RFD) within the first 100 ms had not yet returned to pre-infection levels, indicating a significant deficit. This incomplete recovery might be related to the longer time to functional recovery after neurologic injury and the insufficient intensity of strength training received during recovery. Evidence suggests that a significant proportion of individuals experience symptoms such as fatigue and muscle soreness up to one month following recovery [7, 8, 39–41], which points to the enduring nature of nervous system damage caused by COVID-19. It is noted that even the mildest form of neurological injury, neurapraxia [68] (focal demyelination without damage to the axons or the connective tissues, which can cause decreased nerve conduction velocity and conduction block, resulting in muscle weakness, similar to the damage caused by COVID-19 on nerves)typically requires 3 to 6 months for full structural and functional recuperation [69]. Given that the final testing in this study occurred approximately 35 days post-infection, likely, the nerve’s structure and function were still in the process of healing. The nerve’s capacity for rapid muscle recruitment may not have fully rebounded to its pre-infection state. This could explain why the strength performance, particularly within the initial 200 ms, remained below pre-infection levels after four weeks of recovery.

As previously discussed, cessation of training can cause a reduction in strength and lower limb stiffness [63, 70] and decreases in early RFD and reactive strength performance [20, 67]. In this study, in order to maximize the assurance of the athlete’s health in recovery training, the content and intensity of recovery training were arranged according to the most conservative degree. Athletes ceased strength and specialized training for three weeks following infection. During the fourth and fifth weeks post-infection, they engaged only in low and moderate-intensity strength training and specific training without resuming high-intensity strength training, sprinting, or plyometric training. As noted, the lack of high-intensity strength training could negatively impact the recovery rate of lower limb strength and stiffness in athletes post-COVID-19. This factor may be another reason the early RFD and reactive strength performance have yet to reach pre-infection levels.

In this study, we believe that the decline in strength performance after athletes’ infection is caused by both the novel coronavirus infection and the cessation of training following infection. However, due to the lack of a control group that did not become infected but experienced the same duration of training cessation, we cannot further determine the specific contribution of the above two factors to the decline in strength performance. According to research reports on detraining conditions similar to those in this study, after a 6-week period of strength training cessation, the SJH of athletes decreased by an average of 6.45%, and the CMJH decreased by an average of 5.5% [71]. In contrast, in this study, after only 2 weeks of detraining due to infection, the decrease in SJH (7.71%) and CMJH (9.08%) of the athletes has already far exceeded the results reported in the aforementioned studies. Reports indicate that after 4 weeks of detraining, the DJH of athletes decreased by an average of 7.2% [64]. However, in this study, after 2 weeks of detraining, the athletes experienced a DJH decrease of 18.84%, which is significantly greater than the reduction caused by detraining alone. Reports suggest that after 2–3 weeks of strength detraining, the decrease in athletes’ maximum strength performance is minimal (0.9%-7%) [59, 61]. Yet, in this study, after 2 weeks of detraining following infection, the MIS of the athletes significantly decreased by 18.95%, which is markedly greater than that reported in the aforementioned studies. Additionally, the performance of the early rate of force development (RFD) is primarily related to the recruitment and discharge rate of motor units (MU) [72, 73], and short-term detraining within 2 weeks would not significantly negatively impact the aforementioned performance [59, 73]. However, in this study, after 2 weeks of detraining following infection, the athletes exhibited a substantial decrease in early RFD. By comparing the results of this study with those from similar detraining studies, it can be observed that with less detraining time, the decline in various strength performances of the athletes in this study is several times greater than that of athletes who only underwent detraining. This suggests to some extent that, compared to the impact of post-infection detraining, the damage caused by the novel coronavirus infection may have a more significant effect on the reduction of strength performance and could be the primary factor leading to the significant decrease in strength performance observed in the first test of this study.

In this study, in order to maximize the health status of athletes during the recovery training, the content and intensity of strength and specialized recovery training were arranged at the most conservative level, giving athletes up to 5 weeks to return to their original training level. Some athletes in this study who suffered from severe nerve and muscle damage after infection can safely undergo recovery training in this plan and accumulate some positive adaptation, but at the same time, because the intensity and content of this plan are relatively conservative, it may not be conducive to the rapid recovery of strength for some athletes with relatively mild symptoms. In practice, to better promote the recovery of athletes’ strength, we suggest that strength recovery training should be formulated based on the individual physical condition of the athletes after infection. If the symptoms of neurological and muscular damage in athletes recede quickly, and under the condition of reasonable arrangement of training intensity, resuming strength training as early as possible may be beneficial to maximize the elimination of the adverse effects of detraining on strength performance, and promote a faster recovery of strength performance to the original level. This is very important for athletes who are preparing to participate in competitions shortly after infection.

In summary, the findings of this study demonstrate that athletes experienced a reduction in various aspects of strength performance following COVID-19. The decrease in strength performance may be linked to the negative impact of COVID-19 on both the nervous system and skeletal muscles, as well as the effects of detraining experienced after infection. The slow recovery of reactive strength and early RFD may be due to the gradual neurological recovery process and insufficient high-intensity strength training during the recovery period. However, the current research on the impact of COVID-19 on strength performance is limited, and the specific degree, cause, and mechanism of COVID-19’s impact on strength performance have yet to be reported in detail in other studies. Therefore, the reasons behind the changes observed in strength performance after infection require further verification and study in future research.

Our study has limitations. Because the timing of the athletes’ COVID-19 infection is unknown, we mandated that a pre-infection strength test be conducted on the athletes at most every two months before infection. This resulted in an average interval of about 24 days (23.59±12.51 days) between the pre-infection tests and the actual time of infection with the novel coronavirus. As a result, there is a possibility that our study has underestimated the athletes’ strength performance before infection to some extent, which may lead to an underestimation of the degree of strength decline after infection. Additionally, due to the uncertainty of infection, our study did not establish a control group that was not infected but underwent the same period of training cessation, so we were unable to explore the separate impacts of COVID-19 infection and the subsequent cessation of training on the decline in strength performance.

## Conclusion

In conclusion, this study has shown that following a COVID-19, there is a notable decrease in highly trained athletes’ maximum strength, explosive strength, reactive strength, and early phase force generation capability. After a four-week recovery period, these athletes’ maximum and explosive strengths can nearly revert to their pre-infection states. Nonetheless, the restoration of reactive strength and early phase force generation capability continues to be markedly slower, remaining significantly below the levels observed before infection.
